# Diagnostic Power of MicroRNAs in Melanoma: Integrating Machine Learning for Enhanced Accuracy and Pathway Analysis

**DOI:** 10.1111/jcmm.70367

**Published:** 2025-01-17

**Authors:** Haniyeh Rafiepoor, Alireza Ghorbankhanloo, Soroush Soleimani Dorcheh, Elham Angouraj Taghavi, Alireza Ghanadan, Reza Shirkoohi, Zeinab Aryanian, Saeid Amanpour

**Affiliations:** ^1^ Cancer Biology Research Center, Cancer Institute Tehran University of Medical Sciences Tehran Iran; ^2^ School of Medicine Tehran University of Medical Science Tehran Iran; ^3^ Department of Dermatopathology, Razi Hospital Tehran University of Medical Sciences Tehran Iran; ^4^ Cancer Research Center, Cancer Institute Tehran University of Medical Sciences Tehran Iran; ^5^ Autoimmune Bullous Diseases Research Center, Razi Hospital Tehran University of Medical Sciences Tehran Iran

**Keywords:** bioinformatics, machine learning, melanoma, microRNA

## Abstract

This study identifies microRNAs (miRNAs) with significant discriminatory power in distinguishing melanoma from nevus, notably hsa‐miR‐26a and hsa‐miR‐211, which have exhibited diagnostic potential with accuracy of 81% and 78% respectively. To enhance diagnostic accuracy, we integrated miRNAs into various machine‐learning (ML) models. Incorporating miRNAs with AUC scores above 0.70 significantly improved diagnostic accuracy to 94%, with a sensitivity of 91%. These findings underscore the potential of ML models to leverage miRNA data for enhanced melanoma diagnosis. Additionally, using the miRNet tool, we constructed a network of miRNA–miRNA interactions, revealing 170 key genes in melanoma pathophysiology. Protein–protein interaction network analysis via Cytoscape identified hub genes including MYC, BRCA1, JUN, AURKB, CDKN2A, DDX5, MAPK14, DDX3X, DDX6, FOXM1 and GSK3B. The identification of hub genes and their interactions with miRNAs enhances our understanding of the molecular mechanisms driving melanoma. Pathway enrichment analyses highlighted key pathways associated with differentially expressed miRNAs, including the PI3K/AKT, TGF‐beta signalling pathway and cell cycle regulation. These pathways are implicated in melanoma development and progression, reinforcing the significance of our findings. The functional enrichment of miRNAs suggests their critical role in modulating essential pathways in melanoma, suggesting their potential as therapeutic targets.

## 1 Introduction

Melanoma is responsible for over 75% of deaths related to skin cancer due to its high invasiveness [1]. Despite recent advancements in treating metastatic melanoma, such as immunotherapy, a total of 331,000 new cases of melanoma and over 58,600 associated deaths were recorded, according to the International Agency for Research on Cancer (IARC) in the GLOBOCAN 2022 database [2]. Substantial geographical disparities in incidence were evident across various countries and world regions. Projecting forward, if the rates observed in 2020 persist without significant intervention, it is anticipated that the global burden of melanoma will escalate, reaching an estimated 510,000 new cases and 96,000 deaths by the year 2040 [3]. Melanoma is considered a highly heterogeneous malignancy, with many genetic factors identified since the introduction of next‐generation sequencing. This heterogeneity arises from a combination of environmental exposures, genomic variations, epigenomic modifications and transcriptomic changes [4]. Understanding the interactions among these factors is essential for the early detection of melanoma and can significantly influence mortality rates [5]. Utilising bioinformatics analyses based on genetic and histopathological inputs can aid in classifying and identifying clinically relevant biomarkers, potentially guide management and ultimately improve patient outcomes. Thus, the identification of efficient and accurate diagnostic and prognostic biomarkers, along with their interrelationships, is essential for improving poor prognosis, identifying high‐risk and early‐stage patients, and developing effective treatment strategies. This approach can ultimately reduce melanoma recurrence through noninvasive methods [6, 7]. In metastatic melanoma, there is a pivotal need to discover novel noninvasive biomarkers to enhance various aspects of clinical management, including, staging, risk assessment and prediction of therapeutic responses [8]. MicroRNAs (miRNAs) are a class of small noncoding RNA molecules that play a crucial role in regulating gene expression at the posttranscriptional level, silencing RNA and controlling biological activities such as cell death, proliferation and differentiation. Over recent decades, there has been a significant academic interest in examining changes in miRNA expression patterns within human cancers. Such research aims to clarify the mechanistic roles of miRNAs in cancer initiation and progression, as well as their potential in terms of diagnostic, therapeutic and prognostic targets [9, 10]. Recent studies have shown that changes in miRNA expression may play a role in the progression of skin cancers such as melanoma [11]. The diagnostic and prognostic significance of microRNAs in melanoma has been explored, with substantial support derived from the application of advanced bioinformatics tools [12, 13], but the exact mechanism of how miRNAs affect melanoma growth and metastasis is not fully understood. miRNAs play significant roles in melanoma progression by regulating gene expression within essential signalling pathways like MAPK/ERK, PI3K/AKT and Wnt, through miRNAs such as miR‐21, miR‐214, miR‐203, miR‐211 and let‐7 which influence melanoma's progression, invasiveness and metastatic potential by targeting genes within these pathways [8, 9]. For instance, miR‐21, which is often upregulated in melanoma, targets PTEN, a tumour suppressor that inhibits the PI3K/AKT pathway. By downregulating PTEN, miR‐21 promotes cell survival and proliferation in melanoma, contributing to tumour progression [14, 15]. Similarly, miR‐214 targets CTNNB1, a critical component of the Wnt pathway, and downregulation of CTNNB1 by miR‐214 reduces cell invasiveness and metastatic potential in melanoma [16]. Additionally, miR‐203 and miR‐211 are considerable factors in melanoma's pathophysiology. MiR‐203 functions as a tumour suppressor by targeting ABL1, which is involved in the MAPK pathway [17]. The downregulation of miR‐203 in melanoma has been linked to increased tumour growth and invasiveness [18]. Meanwhile, miR‐211, originating from the *TRPM1* gene, is involved in melanoma cell adaptation to hypoxia by regulating the MITF pathway. This adaptation allows melanoma cells to thrive in hypoxic tumour microenvironments, enhancing metastatic potential [19]. Moreover, the let‐7 family, which targets oncogenes such as RAS, helps suppress the MAPK/ERK pathway when expressed at adequate levels. Lowered let‐7 expression in melanoma cells enables unchecked proliferation and enhanced invasiveness, indicating its essential role in melanoma pathogenesis [20, 21]. Altogether, these miRNAs contribute to melanoma initiation, progression and metastasis by influencing these crucial signalling pathways. It should also be put into consideration that various molecular changes underlie the initiation and progression of melanoma, including the mutations in *BRAF* and *NRAS* genes [22], hyperactivation of the PI3K/AKT pathway [23], inactivation of p53 [24] and alterations in the CDK4/CDKN2A axis [25]. Furthermore, a multitude of studies have elucidated the indispensable contribution of miRNAs to both the initiation and progression phases of melanoma. Hence, miRNAs are regarded as significant regulators of gene expression [26]. This study investigates the diagnostic potential of specific microRNAs in distinguishing melanoma from nevus, highlighting hsa‐miR‐26a and hsa‐miR‐211, which exhibited high discriminatory power with area under the curve (AUC) scores of 81% and 78% respectively. To improve diagnostic accuracy, we integrated these miRNAs into various machine learning (ML) models. The incorporation of miRNAs resulted in a notable increase in diagnostic accuracy, achieving up to 94% with a sensitivity of 91%. Moreover, we constructed a miRNA–miRNA interaction network using the miRNet tool, revealing key regulatory genes in melanoma, including *PLAG1*, *TGFBR2*, *CDKN2A*, *MAPK14* and *MYC*, which exhibited significant degrees and betweenness centrality in the network analysis. The protein–protein interaction (PPI) network analysis identified hub genes such as *MYC*, *BRCA1* and *AURKB*, which play critical roles in melanoma biology. Additionally, gene ontology (GO) and pathway enrichment analyses highlighted the involvement of the PI3K/AKT signalling pathway, TGF‐beta signalling pathway and cell cycle regulation in melanoma progression. Our findings suggest that miRNAs are pivotal in modulating key biological processes in melanoma and may serve as noninvasive biomarkers for early detection and prognosis.

## 2 Methods

### 2.1 Microarray Dataset Search

To identify miRNA expression profiles, we conducted a systematic search in the Gene Expression Omnibus (GEO) database (https://www.ncbi.nlm.nih.gov/geo/) [27]. Using the keywords ‘melanoma’, we reached 59,265 datasets. Then, we limited the results using ‘ *Homo sapiens* ’ and ‘Non‐coding RNA profiling by array’ filters, so we reached 54 datasets. By setting the target population, studies were selected. Ultimately, by considering the adjusted *p*‐value of the microRNAs, seven final datasets were archived.

### 2.2 Differentially Expressed miRNAs (DEMs) Detection

The DEMs were obtained using the online tool GEO2R in the GEO database [27], which makes evaluations using the GEO query and limma R packages from the Bioconductor project to compare two groups of samples in a GEO dataset. Normalisation has been carried out using the RMA algorithm. To keep away from the false‐positive/negative and the differences between microarray platforms, the microarray gene expression profiles of melanoma groups were compared to the normal groups of each dataset separately. Log_2_ (RMA signal intensity fold change) ≥ 0 for upregulation and ≤ 0 for downregulation, and adjusted *p*‐value > 0.05, Benjamini and Hochberg correction, was set as a cut‐off to identify the significant DEMs in GEO datasets. Subsequently, significantly expressed DEMs in each study were listed respectively. Prognostic and diagnostic analysis have been done independently.

### 2.3 Data Integration

A Venn diagram tool from the Bioinformatics & Evolutionary Genomics platform was utilised to integrate all datasets and identify overlapping differentially expressed miRNAs (http://bioinformatics.psb.ugent.be/webtools/Venn/). To enhance the coverage of differentially expressed miRNAs and minimise the risk of overlooking critical genes that may not have demonstrated expression differences in a single study for several reasons, it was determined that only DEMs that overlapped among at least three datasets would be selected for the diagnostic tissue analysis.

### 2.4 Area Under Curve (AUC) Analysis

The expression values of all overlapping DEMs were extracted and analysed using GraphPad Prism software. Following the normalisation of these values, receiver operating characteristic (ROC) curves and the area under the ROC curve (AUC) were employed to evaluate the efficacy of each miRNA in distinguishing melanoma patients from the control group, based on the sensitivity and specificity of each DEMs.

### 2.5 MicroRNA Integration Predictability by Machine Learning (ML) Modelling

Eight machine learning (ML) algorithms—neuronal boosted (NeB), support vector machine (SVM), boosted tree (BT), bootstrap forest (BF), K‐nearest neighbour (KNN), generalised regression lasso (GRL), nominal logistic (NL) and Naïve Bayes (NB)—were employed to develop an optimal predictive model for melanoma diagnosis in order to enhance diagnostic predictability through the integration of overlapping microRNA biomarkers. MicroRNAs exhibiting an area under the curve (AUC) greater than 0.75 across overlapping datasets were selected for model construction. The predictive quality of the machine learning models was evaluated using *R* ^2^ *Y*, which quantifies the convenience of fit and variability explained by the models. The most effective predictive models were identified based on the selection of key distinguishing variables, leading to the maximum value of *R* ^2^ *Y*. The *R* ^2^ *Y* values were calculated and validated using separate training and validation datasets to ensure robustness and reliability in the model performance assessment. Model performance was further assessed through K‐fold cross‐validation and accuracy metrics. To further enhance our analysis, we conducted a predictive partition analysis (PPA). This technique employs a decision tree approach to categorise or forecast data responses based on input variables. PPA is particularly useful for identifying optimal cut‐off points for these variables, allowing us to better understand the relationship between predictors and outcomes. By systematically partitioning the data, the decision tree can reveal how various levels of predictor variables influence the predicted outcomes, providing valuable insights into the decision‐making process. The ML component of the decision tree is focused on determining the appropriate conditions for data partitioning between branches. This partitioning involved dividing the dataset into subsets designated for both training and validation purposes.

### 2.6 MicroRNA–MicroRNA Interactions and Network Analysis

The miRNet tool was employed to identify key genes linked to microRNAs, thereby enhancing the understanding of posttranscriptional regulatory networks in melanoma. From the collection of differentially expressed miRNAs, we selected specific miRNAs for target gene prediction utilising the miRNet database, which is recognised for its aggregation of experimentally validated miRNA targets documented in the literature. The minimum interaction score was established at a confidence level of 0.7, serving as a reliability threshold for the interactions within the network for gene selection, in addition to requiring a degree greater than two.

### 2.7 Gene Ontology (GO) and Pathway Enrichment Analysis

The EnrichR platform leverages the Kyoto Encyclopedia of Genes and Genomes (KEGG) and Reactome databases. These databases house genomic data and biological pathways for comprehensive analysis. Statistical analysis was conducted with a significance threshold set at *p*‐value < 0.05. The enrichment analysis of biological processes was through Metascape (https://metascape.org) [28], which is an online platform specialised in comprehensive gene annotation and analysis resources. Gene ontology (GO) was utilised to identify and describe genes and proteins, facilitating the exploration of the inherent biological properties within the chip database.

### 2.8 Protein–Protein Interaction (PPI) Network Analysis

The Search Tool for the Retrieval of Interacting Genes (STRING) database (https://string‐db.org/) [29] was used to evaluate the PPI network information. The FDR ≤ 0.05 and strength ≥ 0.01, high confidence score (0.700) and the combined score > 0.4 were set as the cut‐off criterion. Next, the PPI network was visualised using Cytoscape (version 3.10.2). The molecular complex detection (MCODE) of Cytoscape was used to analyse modules in the PPI networks with a degree cut‐off = 2, node score cut‐off = 0.2, max depth = 100 and *k*‐score = 2. Functional enrichment analyses of the genes in the modules were performed using KEGG. The degree of each protein node was calculated using a plugin in Cytoscape. In our research, the genes with the degree ≥ 5 were identified as hub genes. A network of genes and their coexpression genes was analysed via GeneMANIA (http://www.genemania.org/) [30, 31], which is a convenient web portal for analysing gene lists and predicting gene function. Finally, Drug–Gene Interaction database (DGIdb) 3.0 (http://www.dgidb.org/) [32] was adopted here to predict drugs based on the module genes. The network map was then formed by Cytoscape.

## 3 Results

### 3.1 Microarray Dataset Search Results

The comprehensive details of the analysed datasets are presented in Table S1. Some of these datasets contain a large set of expression profiles across a range of various cancers, including melanoma. In these studies, a substantial number of healthy controls were incorporated as normal reference groups. Due to the capability of Limma analysis to handle imbalanced data, all control samples were included in the analysis.

### 3.2 Differentially Expressed miRNAs (DEMs) and Overlapped DEMs

A combined total of 127 and 181 miRNAs exhibited significant upregulation and downregulation, respectively, in the diagnostic tissue microarray datasets. In the prognostic datasets, 48 miRNAs were upregulated, and 59 miRNAs were downregulated, showing significant expression changes (Appendix S2). The Venn diagram illustrating the overlap of diagnostic miRNA datasets is presented in Figure S1.

### 3.3 AUC Analysis

Following the extraction of expression values for all selected differentially expressed miRNAs, an analysis of the area under the curve (AUC) was conducted. Subsequently, ROC curves were generated for all DEMs to identify those with the highest discriminatory power in distinguishing melanoma from healthy controls. Finally, nine DEMs showed AUC score > 70% with *p*‐value < 0.01 (Table 1) and their ROC curves are represented in Figure S2. The most promising miRNAs in tissue are hsa‐miR‐26a and hsa‐miR‐211 which were able to distinguish the two groups with 81% (95% CI 0.73–0.88) and 78% (95% CI 0.70–0.85) accuracies.

**TABLE 1:** Expression analysis of differentially expressed miRNAs. AUC, area under curve.

MicroRNAs name	AUC	SE	95% Confidence interval	*p*
**Tissue upregulated miRs (diagnostic)**				
hsa‐miR‐21	0.6002	0.0463	0.5095–0.6908	0.0246
hsa‐miR‐142	0.5446	0.0449	0.4565–0.6327	0.3052
hsa‐miR‐155	0.5062	0.0542	0.3999–0.6125	0.9076
**Tissue downregulated miRs (diagnostic)**				
hsa‐let‐7a	0.5999	0.0523	0.4974–0.7024	0.0398
hsa‐let‐7c	**0.7535**	0.0464	0.6626–0.8445	**< 0.0001**
hsa‐miR‐23b	**0.7379**	0.446	0.6505–0.8254	**< 0.0001**
hsa‐miR‐24	**0.7270**	0.0481	0.6327–0.8213	**< 0.0001**
hsa‐miR‐26a	**0.8056**	0.0377	0.7317–0.8796	**< 0.0001**
hsa‐miR‐26b	**0.7413**	0.0422	0.6584–0.8242	**< 0.0001**
hsa‐miR‐27b	0.5123	0.0544	0.4058–0.6188	0.8045
hsa‐miR‐100	0.5233	0.0609	0.4038–0.6428	0.6861
hsa‐miR‐125b	**0.7636**	0.0395	0.6863–0.8410	**< 0.0001**
hsa‐miR‐139	0.5343	0.0630	0.4108–0.6578	0.5468
hsa‐miR‐193b	**0.7581**	0.0447	0.6705–0.8457	**< 0.0001**
hsa‐miR‐195	**0.7498**	0.0445	0.6625–0.8371	**< 0.0001**
hsa‐miR‐204	0.5892	0.0658	0.4603–0.7182	0.1690
hsa‐miR‐211	**0.7795**	0.0401	0.7009–0.8582	**< 0.0001**
hsa‐miR‐574	0.5269	0.0677	0.3942–0.6596	0.6780
**Tissue downregulated miRs (prognostic)**				
hsa‐miR‐203	0.5808	0.0477	0.4874–0.6742	0.0912
hsa‐miR‐205	0.5902	0.0475	0.4971–0.6833	0.0593
**Tissue upregulated miRs (prognostic)**				
hsa‐miR‐21	0.6022	0.0396	0.5246–0.6797	0.0112

*Note:* The bold numbers showed the significant microRNAs which showed accuracy more than 0.70 and significant p‐value.

### 3.4 MicroRNA Integration Predictability by Machine Learning (ML) Modelling

**FIGURE 1:**
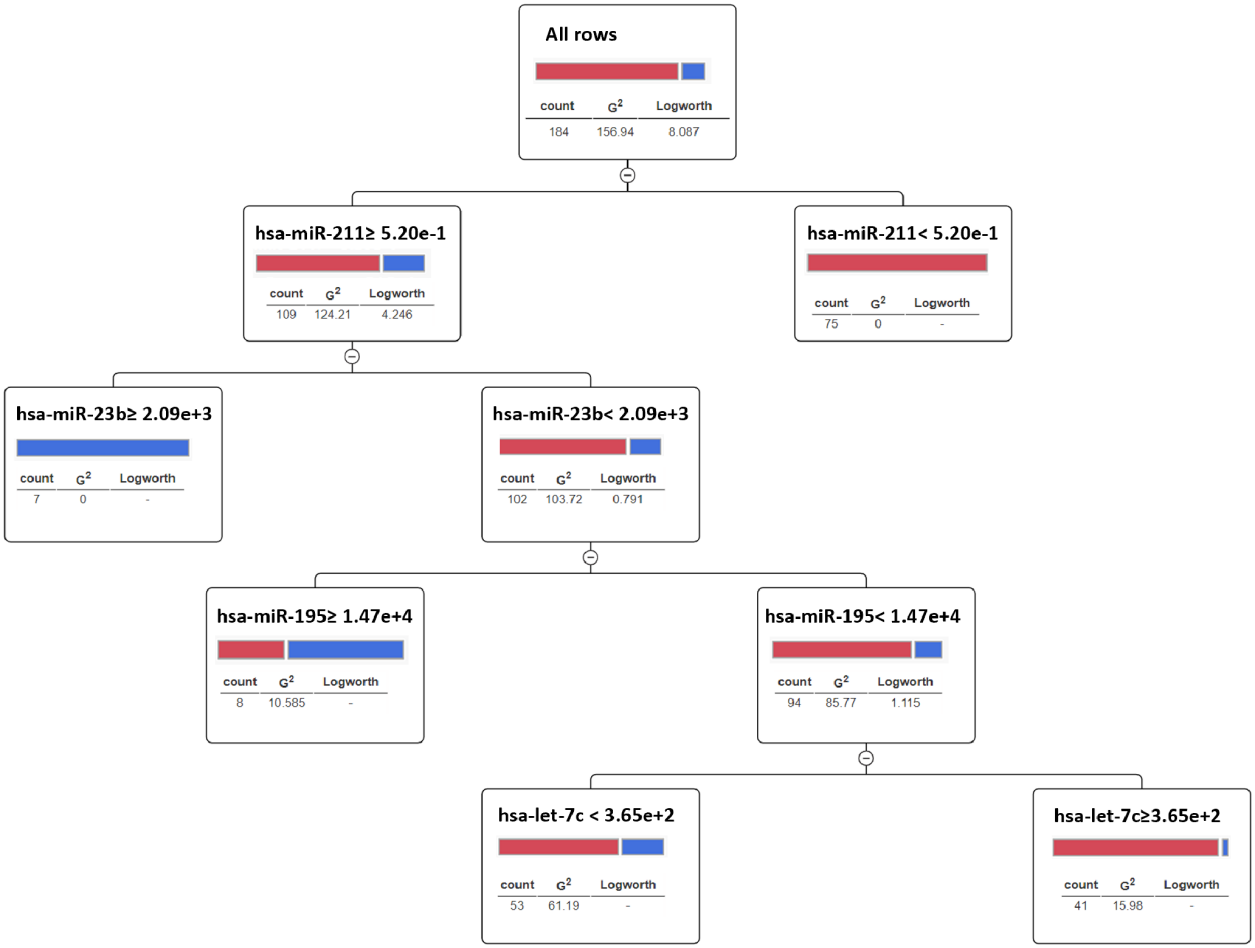
Predictive partition analysis (PPA) shows the most differentiating markers predicting melanoma diagnosis.

The ML‐based approach demonstrated that the integration of microRNAs with the highest predictability (AUC > 0.70) was consistent across at least three common datasets for tissue (hsa‐let‐7c, has‐miR‐23b, has‐miR‐125b, has‐miR‐193b, has‐miR‐195, has‐miR‐211) using different ML approaches, resulting in an accuracy increase up to 0.94 with the sensitivity of 0.91 respectively (Table S2). A PPA analysis was performed to prioritise biomarkers and establish cut‐off values (Figure 1). This analysis highlighted the predictive potential of the combined use of hsa‐miR‐211, hsa‐miR‐23b, hsa‐miR‐195 and hsa‐let‐7c, which yielded an AUC of 0.89. These findings suggest that the integration of a select group of microRNAs can effectively predict cancer diagnosis with a satisfactory level of accuracy.

### 3.5 MicroRNA–MicroRNA Interactions and Network Analysis

**FIGURE 2:**
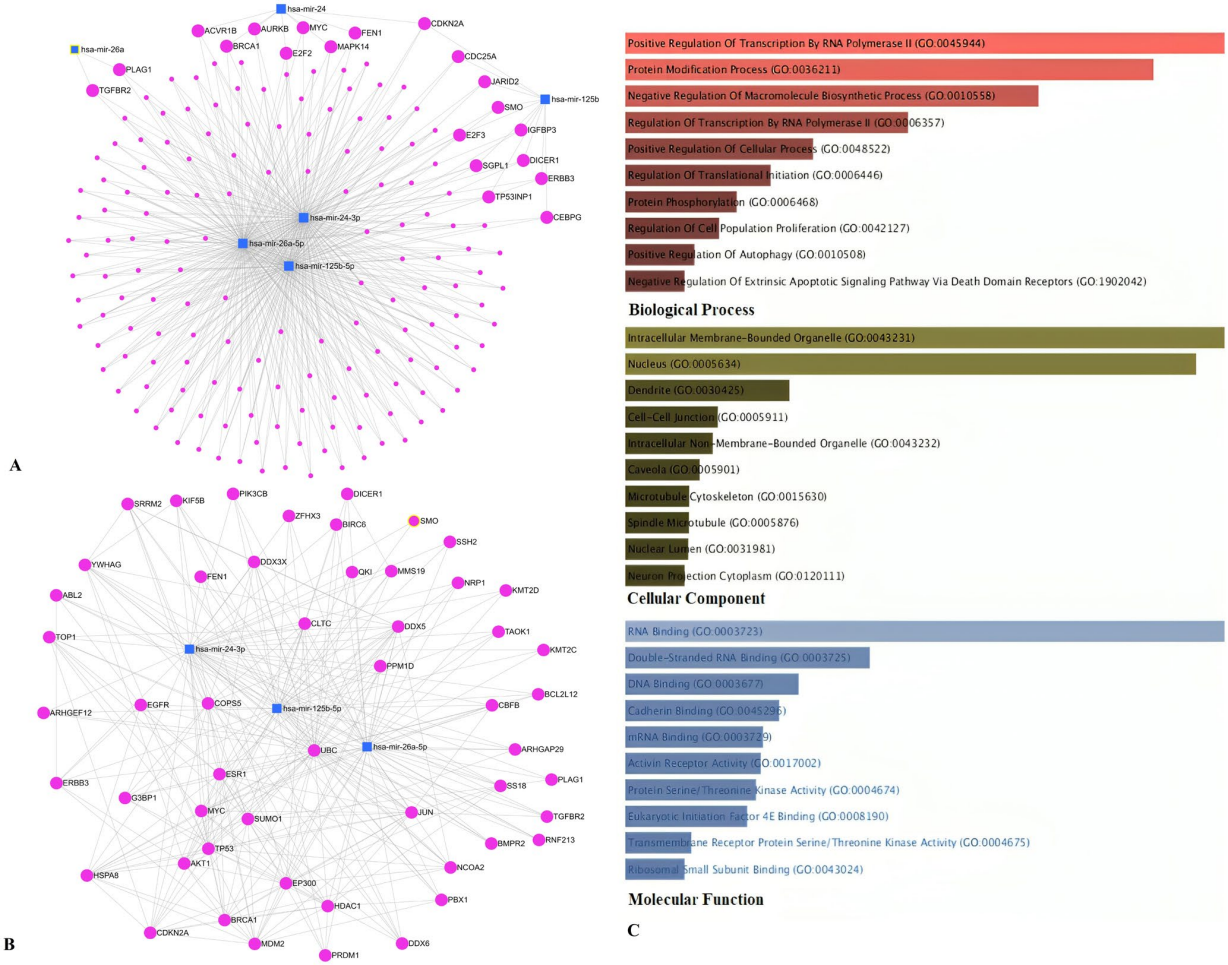
MicroRNAs–genes interaction and enrichment analysis. (A) The miRNA–miRNA interactions all considered microRNAs and related genes. The pink circles represent all predicted target genes, and the blue ones represent the microRNAs implicated in cancer‐related pathways. The lines represent the connection of components. (B) Well‐known target genes for melanoma in associations with the considered differentially expressed miRNAs (DEMs). (C) Gene Ontology (GO) enrichment and KEGG pathway analysis of the differentially expressed genes (DEGs), including biological process (red bars), cellular components (green bars), molecular function (blue bars).

The miRNet network was constructed with 170 genes microRNA nodes and 516 edges, resulting in an average node degree of 5.86. The average betweenness was calculated to be 87.47. *PLAG1*, *TGFBR2*, *CDKN2A*, *MAPK14*, *E2F2*, *E2F3*, *SGPL1*, *TP53INP1*, *AURKB*, *ACVR1B*, *BRCA1*, *FEN1* and *MYC* indicated the highest degree and betweenness among other genes (Figure 2A). The full list of those DEMs in addition to their predicted target genes is available in Appendix S3. A module comprising melanoma‐related genes, as identified from the GDC and TCGA datasets, along with their associated miRNAs, was extracted from this network and is illustrated in Figure 2B.

### 3.6 Gene Ontology (GO) and Pathway Enrichment Analysis

**FIGURE 3:**
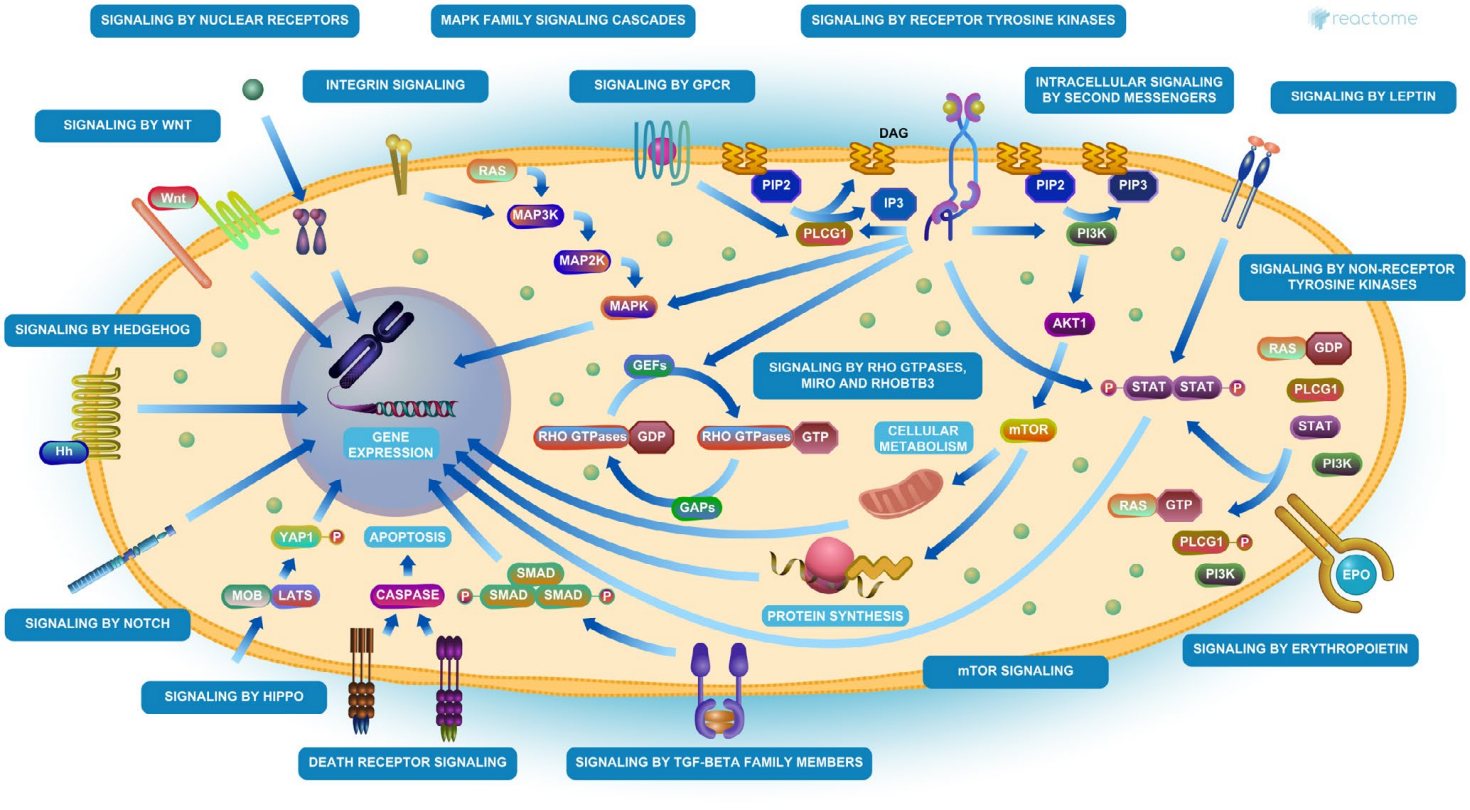
Reactome signal transduction of R‐HSA‐162582 (https://reactome.org/content/detail/R‐HSA‐162582).

To comprehend the differentially expressed genes (DEGs) functional levels, the online biological tool EnrichR was performed using the GO analysis with a significance threshold of *p*‐value < 0.05. The results of the 170 DEGs in the GO terms of the categories were divided into three groups as follows: biological process (BP), cellular component (CC) and molecular function (MF). As indicated in Figure 2C, the CC of overexpressing DEGs was enriched in intracellular membrane‐bounded organelle, nucleus and cell–cell junction. The BP of the overexpressing DEGs was enriched in RNA polymerase II transcription, protein modification and macromolecule biosynthesis. The MF of the overexpressing DEGs was enriched in RNA binding, double‐stranded RNA binding, DNA binding and Cadherin binding. Furthermore, to distinguish the potential pathway from DEGs, we used KEGG pathway enrichment analyses (Table 2). The most important KEGG pathways include cellular senescence, cell cycle, signalling pathways regulating pluripotency of stem cells, ErbB signalling pathway, Hippo signalling pathway, TGF‐beta signalling pathway which some of them were previously known in melanoma pathogenesis. Interestingly, in Reactome 2022 pathway analysis, 53 genes of 170 genes, *DDX5*, *MAPK14*, *TMEM59*, *SOX4*, *SMO*, *ARHGAP29*, *DSG2*, *TAOK1*, *E2F3*, *NRP1*, *ERBB3*, *TNFRSF10B*, *ARF6*, *KMT2D*, *FASN*, *YWHAG*, *KIF5B*, *ADAM17*, *AURKB*, *GSK3B*, *MTMR4*, *SP1*, *SMAD3*, *TGFBR2*, *MYLK*, *POU2F1*, *PEA15*, *RIT1*, *PRDM1*, *SCD*, *JUN*, *PARD6B*, *AGO1*, *BMPR2*, *B4GALT1*, *DIAPH1*, *AKT2*, *PSAP*, *MOB1A*, *ARHGEF12*, *KIF3A*, *NCOA2*, *PBX1*, *CBFB*, *UHMK1*, *EIF4E*, *ABL2*, *ACVR1B*, *MYC*, *CLTC*, *CHD1*, *CHD8* and *PIK3CB*, were involved in signal transduction R‐HSA‐162582, which included some famous pathways such as Hippo, TGF‐beta, WNT and other pathways (Figure 3). The results demonstrated that up‐ and downregulation of microRNA‐related DEGs could have developed melanoma by different carcinogenic pathways which could be modulated by overlapped microRNAs.

**TABLE 2:** Kyoto Encyclopedia of Genes and Genomes (KEGG) and Reactome pathway enrichment analysis.

KEGG 2021 signalling pathways	Adj *p*	Odds ratio	Genes	
Cellular senescence	< 0.0001	8.42	*CDKN2A, MYC, IGFBP3, E2F2, E2F3, PIK3CB, FOXM1, MAPK14, CDC25A, TGFBR2*	
MicroRNAs in cancer	< 0.0001	5.45	*BMPR2, CDKN2A, DICER1, BRCA1, PIK3CB, SLC7A1, CDC25A, GLS, ERBB3, MYC, E2F2, E2F3, SOX4*	
Cell cycle	< 0.0001	8.39	*GSK3B, CDKN2A, MYC, CDC27, E2F2, E2F3, CDC25A, YWHAG*	
Signalling pathways regulating pluripotency of stem cells	0.001	7.20	*GSK3B, ZFHX3, BMPR2, MYC, PIK3CB, JARID2, ACVR1B, MAPK14*	
ErbB signalling pathway	0.002	9.15	*GSK3B, JUN, ERBB3, MYC, ABL2, PIK3CB*	
Hippo signalling pathway	0.005	5.42	*MOB1A, GSK3B, PARD6B, BMPR2, MYC, YWHAG, TGFBR2*	
TGF‐beta signalling pathway	0.010	6.72	*BMPR2, SP1, MYC, ACVR1B, TGFBR2*	
Transcriptional mis‐regulation in cancer	0.010	4.56	*SS18, DDX5, SP1, MYC, IGFBP3, PBX1, TGFBR2*	
AGE‐RAGE signalling pathway in diabetic complications	0.010	6.29	*DIAPH1, JUN, PIK3CB, MAPK14, TGFBR2*	
Proteoglycans in cancer	0.020	4.26	*DDX5, ARHGEF12, SMO, ERBB3, MYC, PIK3CB, MAPK14*	
Lipid and atherosclerosis	0.020	4.05	*GSK3B, JUN, POU2F1, TNFRSF10B, PIK3CB, MAPK14, HSPD1*	
p53 signalling pathway	0.020	6.90	*CDKN2A, IGFBP3, TNFRSF10B, PPM1D*	
Thyroid hormone signalling pathway	0.020	5.15	*GSK3B, NCOA2, MYC, PIK3CB, ATP1B1*	
Sphingolipid metabolism	0.040	7.73	*UGCG, SGPL1, PSAP*	
Progesterone‐mediated oocyte maturation	0.050	4.95	*CDC27, PIK3CB, MAPK14, CDC25A*	
Longevity regulating pathway	0.050	4.85	*EIF4EBP2, PIK3CB, ADIPOR2, APPL1*	
C‐type lectin receptor signalling pathway	0.050	4.75	*JUN, ARHGEF12, PIK3CB, MAPK14*	
T‐cell receptor signalling pathway	0.050	4.75	*GSK3B, JUN, PIK3CB, MAPK14*	
Hedgehog signalling pathway	0.050	6.70	*GSK3B, SMO, KIF3A*	
**Reactome 2022 signalling pathways**	***p***	**Strength**	**Observed gene count**	**Background gene count**
HSA‐162582: Signal transduction	3.88E‐06	0.37	53	2540
HSA‐2262752: Cellular responses to stress	0.00018	0.54	23	747
HSA‐3700989: Transcriptional regulation by TP53	0.00018	0.7	16	361
HSA‐74160: Gene expression (transcription)	0.00018	0.41	34	1476
HSA‐9006931: Signalling by nuclear receptors	0.00038	0.74	13	265
HSA‐212436: Generic transcription pathway	0.0004	0.43	29	1215
HSA‐8939211: ESR‐mediated signalling	0.00044	0.81	11	190
HSA‐9018519: Oestrogen‐dependent gene expression	0.00046	0.93	9	119
HSA‐2559583: Cellular senescence	0.00063	0.84	10	163
HSA‐73857: RNA polymerase II transcription	0.00063	0.4	30	1337
HSA‐1655829: Regulation of cholesterol biosynthesis by SREBP	0.0028	1.09	6	55
HSA‐2559585: Oncogene induced senescence	0.0032	1.23	5	33
HSA‐69278: Cell cycle, mitotic	0.0038	0.53	16	526
HSA‐2559580: Oxidative stress–induced senescence	0.0039	0.93	7	92
HSA‐2426168: Activation of gene expression by SREBF	0.0076	1.13	5	42
HSA‐1640170: Cell cycle	0.0128	0.46	17	658
HSA‐5633007: Regulation of TP53 activity	0.014	0.75	8	159
HSA‐69273: Cyclin A/B1/B2 associated events during G2/M transition	0.014	1.26	4	25
HSA‐69242: S Phase	0.0146	0.74	8	162
HSA‐9006936: Signalling by TGFB family members	0.0146	0.81	7	121
HSA‐5663202: Diseases of signal transduction by growth factor receptors and second messengers	0.0156	0.53	13	430
HSA‐6804756: Regulation of TP53 activity through phosphorylation	0.0218	0.87	6	92

### 3.7 Hub Gene and Protein–Protein Interaction Network Analysis

**FIGURE 4:**
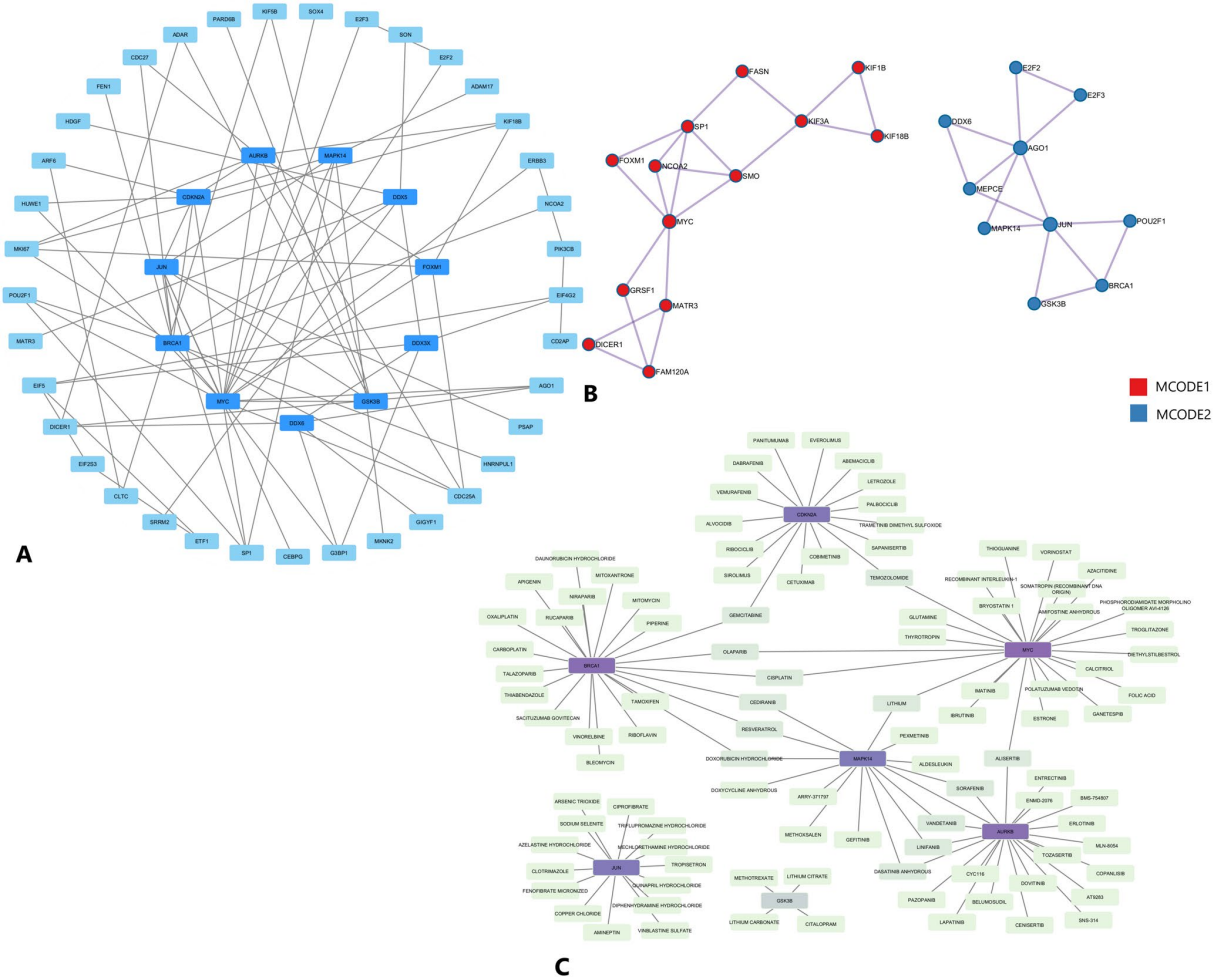
Protein–protein interaction (PPI) network between all of the target genes of considered miRNAs. (A) Common differentially expressed genes (DEGs) and PPI network constructed by Cytoscape database and module analysis, the central circle with darker blue represents the hub genes introduced in this study; (B) the molecular complex detection (MCODE) analysis of top two cluster of highly interconnected genes; (C) 119 drug–gene interaction pairs based on the DGIdb predictions of the module genes, purple square indicates the DEGs and green square indicates the drugs.

For each given 170 gene list, protein–protein interaction enrichment analysis has been conducted with the STRING (physical score > 0.132) and BioGrid database. The resultant network contains the subset of proteins that form physical interactions with at least one other member in the list. The PPI network with combined scores greater than 0.4 and minimum required interaction score greater than 0.7 was generated by Cytoscape, which contained 170 nodes and 92 edges (Figure 4A). Eleven genes with degrees ≥ 5 were identified as hub genes. Detailed information on hub genes, including gene symbols, degrees, betweenness, full names and gene function, is shown in Table 3. The molecular complex detection (MCODE) algorithm has been applied to identify densely connected network components. The MCODE networks identified for individual gene lists have been gathered and are shown in Figure 4B. Pathway and process enrichment analysis has been applied to each MCODE component independently, and the two best‐scoring terms by *p*‐value have been retained as the functional description of the corresponding components. Cluster 1 of MCODE analysis with a score of 1.538 included *KIF1B*, *MYC*, *GRSF1*, *MATR3*, *SMO*, *FAM120A*, *NCOA2*, *SP1*, *KIF3A*, *KIF18B*, *DICER1*, *FOXM1*, *FASN* genes which were mainly involved in PID FOXM1 pathway and activation of gene expression by *SREBF* (SREBP) with −6.2 Log_10_(*p*). Cluster 2 with a score of 1.5 included *E2F3*, *GSK3B*, *JUN*, *BRCA1*, *MAPK14*, *E2F2*, *MEPCE*, *POU2F1*, *AGO1*, *DDX6*, *RC3H2*, *AURKB*, *DDX3X*, *LARP1* genes played a role in oxidative stress–induced senescence with −9.6 Log_10_(*p*). Network of the hub genes and their coexpression genes was analysed by GeneMANIA online platform. The 11 genes showed the complex PPI network with the coexpression of 28.42%, physical interactions of 39.57% colocalisation of 7.21%, shared protein domains of 9.39% and predicted of 1.10% (Figure S3). Lastly, based on the DGIdb predictions of the hub genes, we obtained 443 drug–gene interaction pairs (119 FDA‐approved), including seven genes (*BRECA1*, *MYC*, *CDKN2A*, *MAPK14*, *JUN*, *GSK3B* and *AURKB*) as shown in Figure 4C. These results may reveal the therapeutic targets related to melanoma.

**TABLE 3:** Top hub genes with the highest degree and their function.

Gene symbol	Degree	Description	Function
*MYC*	17	*MYC* proto‐oncogene, bHLH transcription factor	Involved in cell cycle progression, apoptosis, cellular transformation and transcription of specific target genes
*BRCA1*	11	*BRCA1* DNA repair associated	Helps maintain genomic stability and acts as a tumour suppressor. It forms a complex with other tumour suppressors, DNA damage sensors and signal transducers and also plays a role in transcription, DNA repair of double‐stranded breaks and recombination
*JUN*	8	Jun proto‐oncogene, AP‐1 transcription factor subunit	Interacts with specific target DNA sequences to regulate gene expression
*AURKB*	7	aurora kinase B	Regulation of chromosome alignment and segregation during mitosis and meiosis by interacting with microtubules
*CDKN2A*	7	Cyclin‐dependent kinase inhibitor 2A	Involved in tumour suppression and cell cycle control
*DDX5*	6	DEAD‐box helicase 5	Involved in pathways that alter RNA structures, regulate transcription, splicing and process small noncoding RNAs
*MAPK14*	6	mitogen‐activated protein kinase 14	Involved in various cellular processes such as proliferation, differentiation, transcription regulation, development, stress‐related transcription, cell cycle regulation and genotoxic stress response
*DDX3X*	5	DEAD‐box helicase 3 X‐linked	Involved in transcriptional regulation, mRNP assembly, pre‐mRNA splicing, mRNA export, translation, cellular signalling and viral replication
*DDX6*	5	DEAD‐box helicase 6	Involved in suppressing translation and degrading mRNA, and microRNA‐induced gene silencing
*FOXM1*	5	forkhead box M1	Involved in cell proliferation
*GSK3B*	5	glycogen synthase kinase 3 beta	Involved in various cellular processes such as energy metabolism, inflammation, ER stress, mitochondrial dysfunction and apoptosis

## 4 Discussion

This study identified several miRNAs with significant ability to differentiate melanoma from nevus. Notably, hsa‐miR‐26a and hsa‐miR‐211 demonstrated high diagnostic potential, with area under the curve (AUC) scores of 81% and 78% respectively. To enhance diagnostic accuracy, we assessed the integration of the identified miRNAs into various machine learning (ML) models. Nine ML methods, including XGBoost, support vector machine (SVM) and neural networks were employed to develop prediction models. Integrating miRNAs with AUC scores above 0.70 (including hsa‐let‐7c, hsa‐miR‐23b, hsa‐miR‐125b, hsa‐miR‐193b, hsa‐miR‐195 and hsa‐miR‐211) into these models substantially enhanced diagnostic accuracy to 94%, achieving a sensitivity of 91%. These findings highlight the potential of machine learning models in utilising miRNA data to enhance melanoma diagnosis. Additionally, we employed the miRNet tool to construct a network of miRNA–mRNA interactions, providing insights into the regulatory networks in melanoma. The network analysis identified key genes such as *PLAG1*, *TGFBR2*, *CDKN2A*, *MAPK14*, *E2F2*, *E2F3*, *SGPL1*, *TP53INP1*, *AURKB*, *ACVR1B*, *BRCA1*, *FEN1* and *MYC* highlighted by their high degrees and betweenness centrality, underscoring their essential roles in melanoma biology. Additionally, the PPI network analysis using Cytoscape suggested hub genes including *MYC*, *BRCA1*, *JUN*, *AURKB*, *CDKN2A*, *DDX5*, *MAPK14*, *DDX3X*, *DDX6*, *FOXM1* and *GSK3B*. The identification of these hub genes and their interactions with miRNAs provides a deeper understanding of the molecular mechanisms driving melanoma. The gene ontology (GO) and pathway enrichment analyses further elucidated the biological processes and pathways associated with the differentially expressed miRNAs. Key pathways identified include the phosphatidylinositol‐3′‐kinases PI3K/AKT signalling pathway, TGF‐beta signalling pathway and cell cycle regulation. These pathways are crucially implicated in melanoma development and progression, thereby reinforcing the validity of our findings [33]. The proto‐oncogene *c‐Myc* plays a crucial role in cellular transformation and the regulation of programmed cell death. Clinical and pathological studies have established a positive correlation between *c‐Myc* expression and the occurrence of vasculogenic mimicry (VM), linearly patterned programmed cell necrosis (LPPCN), as well as enhanced cell motility and invasiveness [34]. Additionally, c‐Myc facilitates the expression of the epithelial–mesenchymal transition (EMT)–associated protein Snail. Under hypoxic conditions, elevated c‐Myc levels also lead to increased expression of Bax, promoting apoptosis [35]. The *MYC* gene is implicated in several signalling pathways, including the Hippo pathway, where it is subject to posttranscriptional upregulation through YAP‐mediated downregulation of microRNAs that modulate MYC expression [36]. Glycogen synthase kinase 3 (GSK‐3), a serine/threonine kinase, is commonly activated through the PI3K/PTEN/AKT/GSK‐3/mTORC1 pathway, where AKT phosphorylation inhibits GSK‐3 activity. GSK‐3 also interacts with the WNT/β‐catenin signalling pathway, modulating β‐catenin and other key components [37, 38]. Moreover, GSK‐3 influences NF‐κB activity, which is often elevated in cancerous cells. Recent studies indicate that YAP enhances Wnt/β‐catenin signalling by inhibiting GSK‐3β, thereby stabilising β‐catenin [39]. Xin et al. [40] demonstrated that YAP could activate the insulin‐like growth factor (IGF) pathway, as overexpression of a constitutively active form of YAP increased the transcription of IGF pathway‐related genes like IGF1 and β‐catenin. This action enables YAP to drive a β‐catenin transcriptional programme through the IGF pathway independently of Wnt signalling. Both c‐Jun and c‐Myc are unstable oncogenic transcription factors subjected to polyubiquitination. Specifically, GSK‐3 phosphorylates c‐Jun, which enables recognition by the E3 ligase Fbw7, leading to c‐Jun's polyubiquitination and degradation by the proteasome. An inverse relationship exists between GSK‐3 activity and c‐Jun levels throughout the cell cycle, as phosphorylation by GSK‐3 at Ser‐243 requires prior priming. Mutations at this site, such as phenylalanine substitution in v‐Jun, prevent recognition by Fbw7, thereby stabilising v‐Jun and enhancing its oncogenic potential. This highlights the collaborative role of GSK‐3 and Fbw7 in regulating both c‐Jun and c‐Myc stability, a mechanism potentially exploitable in cancer therapy [41]. The forkhead box protein M1 (FOXM1) is another pivotal factor in cell proliferation and DNA repair, with its overexpression being strongly associated with tumour progression in multiple cancers, including cutaneous melanoma [42]. It also modulates the MAPK pathway and interacts with the AKT pathway, thereby facilitating melanoma progression [43]. Further insights into RNA helicases reveal the role of DDX3X in enhancing the translation of MITF, a transcription factor that suppresses melanoma invasiveness when highly expressed. Loss of DDX3X disrupts MITF regulation, prompting melanoma cells to transition from a proliferative to a more invasive, metastatic phenotype [44, 45]. Additionally, DDX3X deficiency triggers aberrant activation of NF‐κB and MAPK pathways, dysregulating cell cycle control and promoting tumour aggressiveness [46]. Mutations in DDX3X also correlate with increased phosphorylation of STAT3 and p42/p44 MAPK, contributing to chemoresistance in melanoma [47]. Other RNA helicases, such as DDX5 and DDX17, are involved in diverse aspects of RNA metabolism, including mRNA splicing, miRNA and ribosome biogenesis, mRNA degradation and transcriptional regulation. These helicases undergo multiple posttranslational modifications—such as phosphorylation, acetylation, ubiquitination and sumoylation—that enhance their versatility in tumorigenesis and tumour progression, underscoring their potential as targets in cancer therapy [48].

The functional enrichment of miRNAs in these pathways suggests that miRNAs play a critical role in modulating key biological processes in melanoma, making them potential targets for therapeutic intervention. Numerous studies have underscored the pivotal function of microRNAs in the onset and advancement of melanoma [26]. The current investigation identifies nine specific miRNAs—hsa‐let‐7c, hsa‐miR‐23b, hsa‐miR‐24, hsa‐miR‐26a, hsa‐miR‐26b, hsa‐miR‐125b, hsa‐miR‐193b, hsa‐miR‐195 and hsa‐miR‐211—as significant contributors to melanoma progression among the hundreds of miRNAs evaluated. miR‐26a is downregulated in melanoma, where it normally acts as a tumour suppressor by targeting EZH2, an oncogenic histone methyltransferase. The loss of miR‐26a in melanoma enhances cell cycle progression, and EZH2 overexpression is associated with more invasive and aggressive tumour phenotypes. The miR‐26a/EZH2 axis plays a significant role in maintaining cell cycle checkpoints, and its disruption is a key event in melanoma's pathogenesis and aggressiveness [49]. Also, hsa‐miR‐26a is previously demonstrated to inhibit proliferation by targeting MITF [50] and induce apoptosis through SODD [51]. This research further elucidates that hsa‐miR‐26a impacts several hub genes, including *MYC*, *BRCA1*, *JUN*, *DDX5*, *MAPK14*, *DDX3X*, *DDX6*, *FOXM1* and *GSK3B* via the p53, Hippo, FoxO and PI3K signalling pathways. Additionally, hsa‐miR‐26b was found to influence the MAPK pathway through TRAF5 [49]. MiR‐125b shows tumour‐suppressive roles, though its function can vary depending on the type of cancer. In melanoma, miR‐125b primarily inhibits apoptosis by targeting pro‐apoptotic genes, including BAK1 and p53, which are vital in controlling cell death pathways. Upregulation of miR‐125b may lead to chemoresistance by helping melanoma cells evade apoptosis [52]. Furthermore, hsa‐miR‐125b was shown to diminish melanoma proliferation and invasion by modulating signalling pathways that target MLK3 [53] and *c‐Jun* genes [54]. This variation in function highlights its complex role in melanoma, where both overexpression and suppression can impact tumour behaviour and treatment outcomes. In this study, it has been concluded that other Jun pathway‐related genes such as MAPK families and GSK3B are also affected by miR‐125b. Previous research indicated that hsa‐miR‐211 exhibits dual expression patterns; its overexpression correlates with reduced proliferation, invasion and tumorigenicity while inhibiting PI3K signalling [19]. Conversely, its downregulation affects growth and invasion by targeting KCNMA1 [55], IGF2R [56] and BRN2 [57], while also regulating cell proliferation through PDK4 [58] and influencing the epithelial–mesenchymal transition (EMT) process via RAB22A [59]. In melanotic melanoma cells, miR‐211 acts as a direct target of MITF and regulates EDEM1 expression and cell migration, subsequently impairing the degradation of Tyrosinase. Thus, miR‐211 facilitates the pro‐pigmentation effects of vemurafenib [60]. The let‐7 family of miRNAs is frequently downregulated in melanoma, where it targets multiple oncogenes, including *NRAS* and *MYC*. These miRNAs are critical for maintaining normal cellular differentiation and preventing unrestrained proliferation [21]. Reduced let‐7 levels lead to the activation of pathways like the MAPK and PI3K/AKT pathways, which support tumour growth and survival. Additionally, low let‐7 levels contribute to treatment resistance, as these miRNAs are involved in regulating cell cycle and apoptosis. Thus, the let‐7 family's downregulation removes a natural brake on oncogenic signalling, contributing to melanoma progression [61]. Let‐7 family have been reported to activate gene translation when the cell cycle is arrested, despite their predominant inhibitory role during cell proliferation. This family is also involved in regulating key cell cycle proteins, including cyclins D1, D3 and A, as well as CDK4. These observations imply that the downregulation of let‐7 miRNAs in melanoma may disrupt cell cycle regulation—a characteristic feature of cancer development and progression. MiR‐193b functions as a tumour suppressor in melanoma, where its downregulation is associated with enhanced tumour cell proliferation and invasiveness. This miRNA targets genes like cyclin D1, inhibiting pathways involved in cell cycle progression. Low levels of miR‐193b have been correlated with poor prognosis and advanced disease stages in melanoma, as this miRNA restricts both cell division and metastatic potential through its modulation of the cyclin‐dependent kinase pathway and the MAPK pathway, both critical in melanoma pathogenesis [62]. Additionally, *Mcl‐2* and *CCND1* [63] are two genes influenced by the downregulation of miR‐193b in melanoma progression. MiR‐195 is another tumour‐suppressive miRNA in melanoma. It targets genes such as *BCL2*, thereby inhibiting cell survival and inducing apoptosis. Downregulation of miR‐195 is frequently observed in melanoma cases with aggressive features, as the loss of this miRNA removes constraints on pathways promoting cell proliferation and resistance to cell death. miR‐195's suppression is also linked to increased tumour progression through its interaction with the PI3K/AKT and MAPK pathways [64]. Although hsa‐miR‐195 has demonstrated prognostic significance with high accuracy in predicting overall survival [65], this study reveals its diagnostic potential. These miRNAs interact with major signalling pathways such as MAPK and PI3K/AKT. For instance, miR‐193b, miR‐125b and the let‐7 family collectively suppress pathways promoting tumour cell survival and proliferation. Dysregulation across these miRNA network allows melanoma cells to bypass cell cycle checkpoints, evade apoptosis and develop resistance to therapy, especially in aggressive melanoma phenotypes [26]. Another noteworthy miRNA is hsa‐miR‐24; its overexpression promotes autophagy and apoptosis in melanoma cells by targeting UBD and activating JNK signalling [66]. The involvement of NF‐kB pathways through NAMPT genes is also highlighted within the context of melanoma pathophysiology [67]. Several bioinformatics studies have employed similar methodologies for identifying cancer genomic profiles, with a primary focus on gene‐level expression in melanoma [68], pancreatic cancer [69] and gastric cancer [70, 71]. Earlier research indicated that the downregulation of miR‐125b [72] and miR‐211 [73] could predict melanoma diagnosis with accuracies of 88% and 96% respectively. However, in this study, these microRNAs demonstrated lower prediction accuracies of 75% and 78%. Nevertheless, by integrating these microRNAs with appropriate machine learning (ML) algorithms, the accuracy can be enhanced to 94%, achieving a sensitivity of 91%. This underscores the significance of microRNA interactions in cancer prediction and highlights the necessity for selecting suitable algorithms to improve model performance. As discussed in previous studies [74, 75, 76], the choice of ML algorithms depends on various factors, including sample size, feature types and assessment needs. The machine learning models employed in this study have been previously applied in bioinformatics and are recognised as established algorithms in the literature [77, 78]. For instance, support vector machines (SVM) are regarded as a prominent tool for marker analysis, demonstrating lower prediction errors compared to classifiers based on alternative methodologies, such as artificial neural networks, particularly when dealing with large feature sets. Additionally, methods like bootstrap forest and decision trees are acknowledged as robust classifiers capable of addressing class imbalance challenges. Decision trees have been extensively utilised for identifying marker cut‐off points. Research has also explored various model assumptions for optimally combining markers linearly into a single composite marker to predict binary disease outcomes [79, 80, 81]. However, linear combinations may lack the necessary flexibility and contextual relevance when integrating markers from diverse domains, whereas nonlinear combinations utilising tree structures offer greater flexibility and interpretability. It is worth mentioning that tree‐based models can manage correlations among markers in a more nonparametric and adaptable manner compared to linear frameworks [82]. Cytoscape is utilised in this study for its powerful visualisation features, the ability to extend functionality through plugins, integration of diverse biological data types and the support of a strong community. Other similar tools exist, such as NetworkX which is excellent for custom analyses. However, Cytoscape's comprehensive functionalities make it especially effective for complex network analyses in systems biology. Its user‐friendly graphical interface allows researchers to easily create, manipulate and explore networks like PPIs and gene regulatory pathways. Unlike earlier research, this study emphasises both microRNA selection and model screening methods to identify the most effective algorithms for model development. Furthermore, the progression from microRNA panels to gene identification and subsequently to associated pathways provides a comprehensive overview of melanoma. The main innovation of this study lies in the integration of diverse datasets to enhance the validity of the findings, coupled with bioinformatic methodologies aimed at comprehensively analysing the melanoma landscape, from microRNAs to genes and proteins, as well as recommending drug‐related markers. Additionally, it is important to highlight that machine learning models have been employed to improve the predictability of these markers that has not been addressed in prior similar research. We have applied this same methodology to prognostic microRNAs. This integrated approach not only enhances the predictive capabilities but also contributes to a deeper understanding of the molecular mechanisms underlying melanoma, paving the way for improved therapeutic strategies. Conversely, employing partition analysis enables us to identify the specific cut‐off values for each microRNA. This identification is crucial, as it holds significant potential for enhancing clinical decision‐making processes in future medical practices. By establishing these thresholds, clinicians can better stratify patients based on their microRNA profiles, ultimately leading to more personalised treatment strategies and improved patient outcomes. Furthermore, this analytical approach may facilitate the development of diagnostic tools that leverage microRNA levels as biomarkers for various diseases, thereby contributing to more effective and targeted therapeutic interventions. These approaches build upon previous studies aimed at developing computational models in the field of microRNAs [83]. These studies have focused on miRNA–disease associations (MDAs) and have introduced at least 29 state‐of‐the‐art models for predicting these associations [77, 78]. In this context, extensive datasets have been developed, including miRBase, miRGator and miRGen, which serve as the foundation for the creation of multifaceted tools such as mirNet [84]. This study has some limitations. Notably, the variability in the number of cases and controls across different studies may introduce biases into the analytical outcomes, although the Limma analysis framework is adept at managing such discrepancies through statistical adjustments [50]. Furthermore, the absence of experimental validation raises questions about the reliability of the findings. Future research is essential to rigorously assess the diagnostic efficacy of the identified biomarkers. Additionally, there is a pressing need for validation studies focusing on gene‐related analyses, particularly concerning the hub genes elucidated in this investigation. Such studies would enhance the robustness of the conclusions drawn and provide a more comprehensive understanding of the underlying biological mechanisms.

In conclusion, this study reinforces the validity of previously identified biomarkers for melanoma while also proposing new candidate microRNAs that could serve as diagnostic or prognostic tools, as well as therapeutic targets. We have developed eight machine learning diagnostic models that incorporate combinations of microRNAs based on their significant expression profiles and functional characteristics. The primary objective of this research was to identify suitable miRNA biomarkers in tissue samples capable of distinguishing between melanoma and nevus, as well as between early and advanced stages of melanoma. Additionally, through the analysis of miRNA interactions, we identified several hub genes and their associated pathways to enhance our understanding of melanoma's pathophysiology. The use of the DGIdb facilitated the identification of potential therapeutic targets in melanoma treatment. This method can also serve as a framework for future studies aimed at developing novel integration models that take into account the specific pathophysiology and histology of complex diseases. However, it is important to note that none of these findings have yet been validated through experimental studies, necessitating further validation in such investigations. While this bioinformatics study has introduced additional biomarkers or panels for consideration in future research, the analyses conducted on these datasets do not currently support the clinical application of these biomarkers without more extensive testing in large‐scale case–control and cohort studies.

## Author Contributions

**Haniyeh Rafiepoor:** conceptualization (equal), formal analysis (lead), methodology (lead), software (equal), validation (equal), visualization (equal), writing – original draft (equal). **Alireza Ghorbankhanloo:** conceptualization (equal), formal analysis (equal), methodology (equal), software (lead), validation (equal), visualization (equal), writing – original draft (lead). **Soroush Soleimani Dorcheh:** writing – original draft (equal). **Elham Angouraj Taghavi:** writing – original draft (equal). **Alireza Ghanadan:** supervision (equal), writing – review and editing (equal). **Reza Shirkoohi:** supervision (equal), writing – review and editing (equal). **Zeinab Aryanian:** investigation (equal), supervision (equal), writing – review and editing (equal). **Saeid Amanpour:** project administration (equal).

## Conflicts of Interest

The authors declare no conflicts of interest.

## Supporting information


Appendix S1

Appendix S2

Appendix S3

## Data Availability

Publicly available datasets were analyzed in this study. This data can be found here: GEO database (https://www.ncbi.nlm.nih.gov/geo), accession numbers: GSE211098, GSE183116, GSE62372, GSE34460, GSE35579, GSE24996, GSE19387.
