# Supplementary material for: Diagnostic Power of MicroRNAs in Melanoma: Integrating Machine Learning for Enhanced Accuracy and Pathway Analysis
Source: J Cell Mol Med. 2025 Jan 17;29(2):e70367. doi: 10.1111/jcmm.70367 (PMC11740884; doi:10.1111/jcmm.70367)
Supplement: Supplementary file 1 — Appendix S1 [file JCMM-29-e70367-s002.docx]

| GSE codes | | Controls/ Cases | Diagnosis /prognosis | Type of samples |
| --- | --- | --- | --- | --- |
| 1 | GSE211098 | Non-ulcerated /ulcerated cutaneous melanoma | Prognosis  (ulceration) | Tissue |
| 2 | GSE183116 | Nevus/ melanoma | Diagnosis | Tissue |
| 3 | GSE62372 | Melanoma/ Metastatic melanoma | Prognosis | Tissue |
|  |  | Nevus/ melanoma | Diagnosis |  |
| 4 | GSE34460 | Melanoma/ Metastatic melanoma | Prognosis | Tissue |
|  |  | Nevus/ melanoma | Diagnosis |  |
| 5 | GSE35579 | Melanoma/ Metastatic melanoma | Prognosis | Cell line and tissue |
|  |  | Nevus/ melanoma | Diagnosis |  |
| 6 | GSE24996 | Melanoma/ Metastatic melanoma | Prognosis | Tissue (FFEP) |
|  |  | Nevus/ melanoma | Diagnosis |  |
| 7 | GSE19387 | Melanoma/ Metastatic melanoma | Prognosis | Tissue |
|  |  | Nevus/ melanoma | Diagnosis |  |
| *Gray datasets are excluded  Only studies with control and case groups considered | | | | |

**Table S1.** Description of assessed datasets

| **Tissue datasets** | | | | | |  |
| --- | --- | --- | --- | --- | --- | --- |
| **Method** | **RSquare** | **Mean RASE** | **StdDev RASE** | **Mean AUC** | **Mean MR** | **Sensitivity** |
| Bootstrap Forest | 0.4268 | 0.27394 | 0.06892 | 0.9420 | 0.11500 | 0.9130 |
| Neural Boosted | 0.4201 | 0.24982 | 0.04805 | 0.8868 | 0.07611 | 0.9782 |
| Boosted Tree | 0.3971 | 0.26395 | 0.09036 | 0.9242 | 0.09389 | 0.9130 |
| K Nearest Neighbors | 0.3789 | . | . | . | 0.05444 | 0.9782 |
| Support Vector Machines | 0.3596 | 0.25660 | 0.04262 | 0.7670 | 0.07056 | 0.9782 |
| Generalized Regression Lasso | 0.1835 | 0.30882 | 0.04938 | 0.7899 | 0.12556 | 1.000 |
| Nominal Logistic | 0.1340 | 0.32134 | 0.05005 | 0.7749 | 0.14667 | 0.9130 |
| Naive Bayes | -1.915 | 0.42046 | 0.07519 | 0.6810 | 0.19278 | 0.7826 |

**Table S2.** Model screening of prediction mortality of gastric cancer patients shows a high AUC and specificity for most ML-based methods such as support vector machine (SVM), neural boosted (NB) and K Nearest Neighbors (KNN). RASE: root average squared error


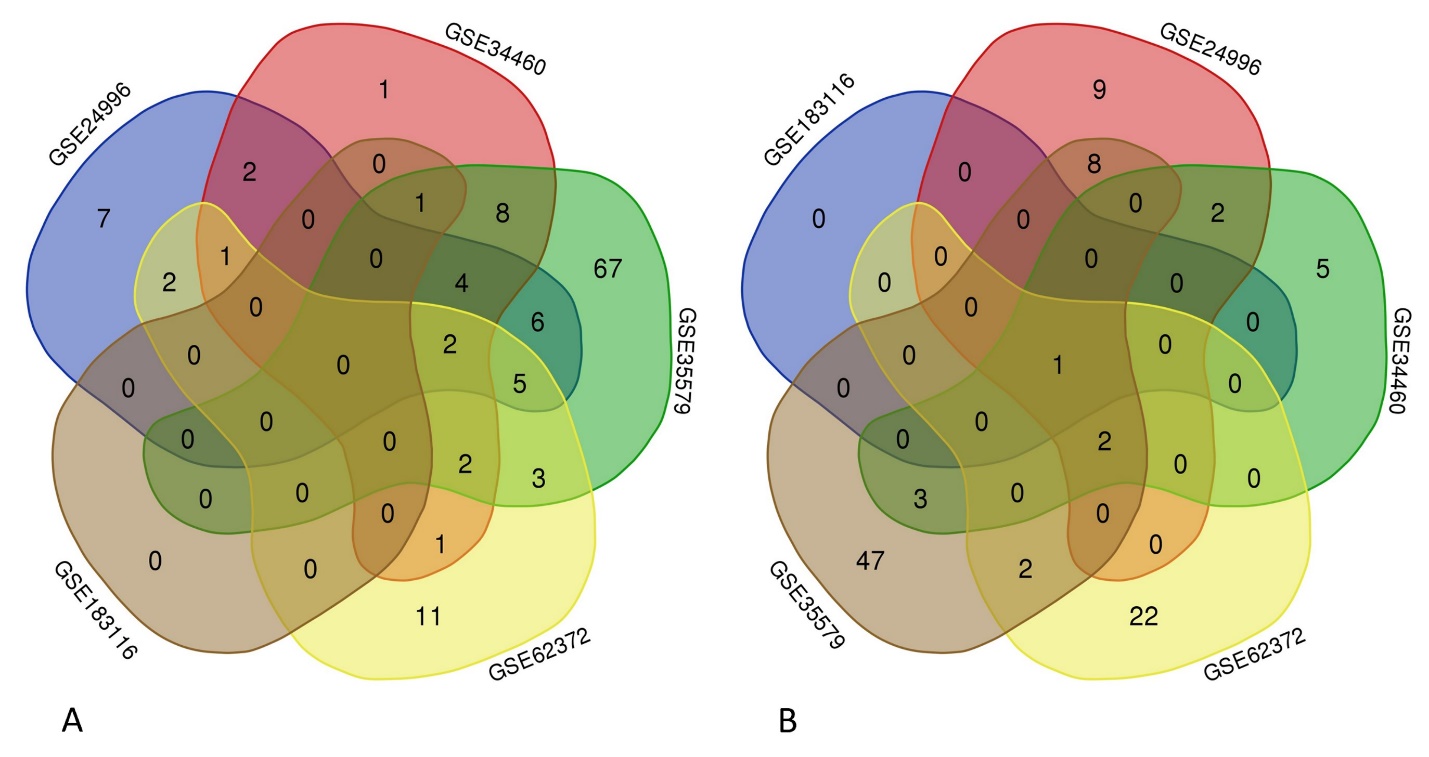


**Figure S1.** A VENN diagram representing the number of DEMs that are in common between different analyzed datasets. The selected diagnostic DEMs (A. Tissue down regulated B. Tissue up regulated)


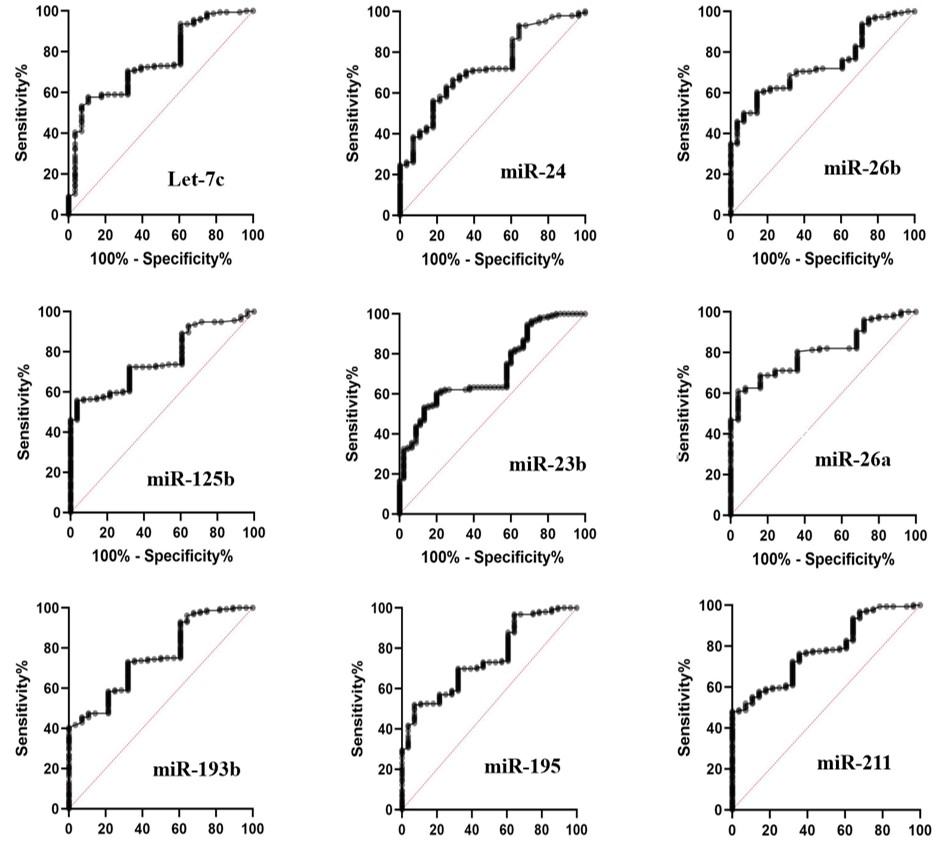


**Figure S2.** The ROC curves for DEMs with AUC>70%


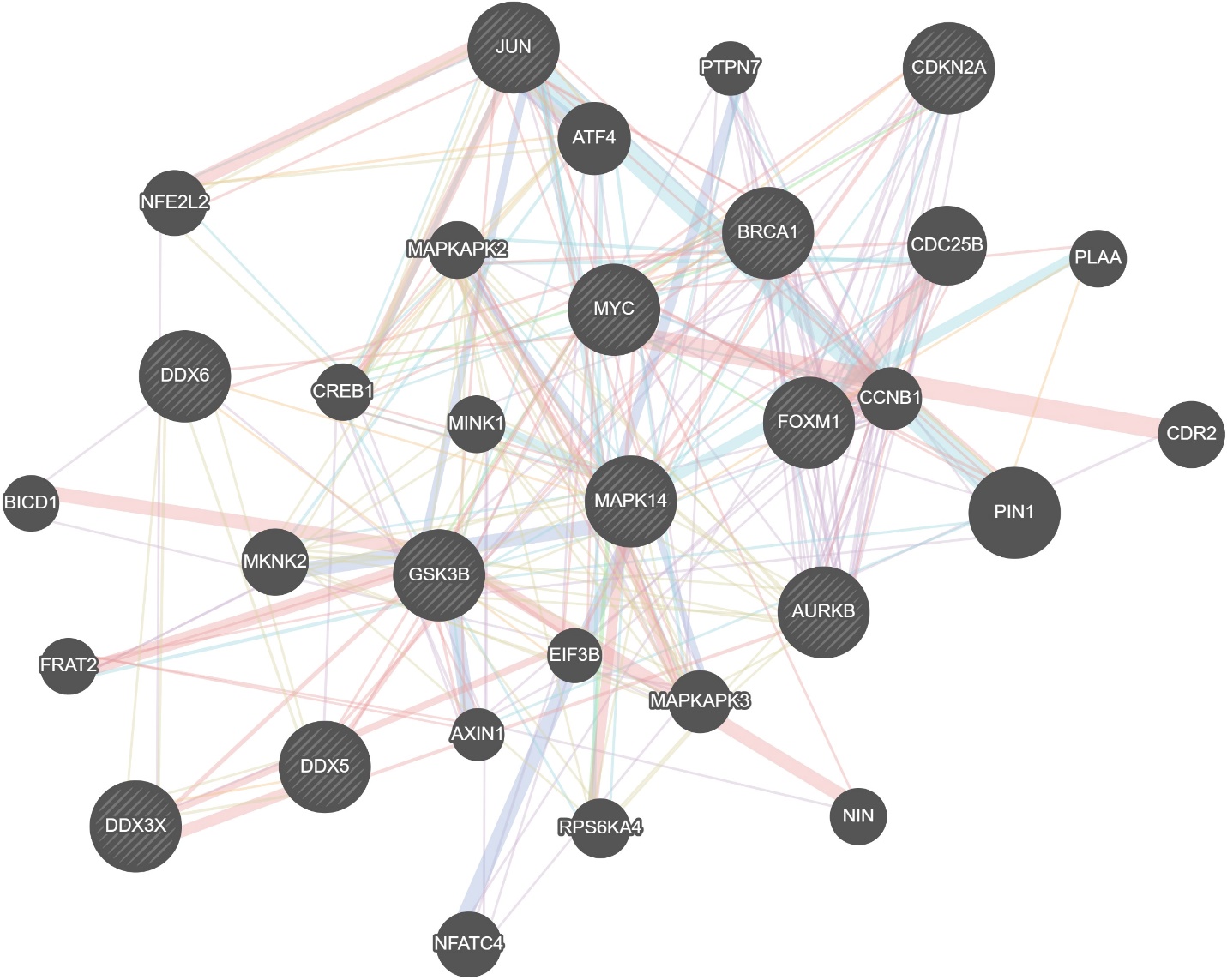


**Figure S3.** Network of the hub genes and their co-expression genes was analyzed by GeneMANIA online platform


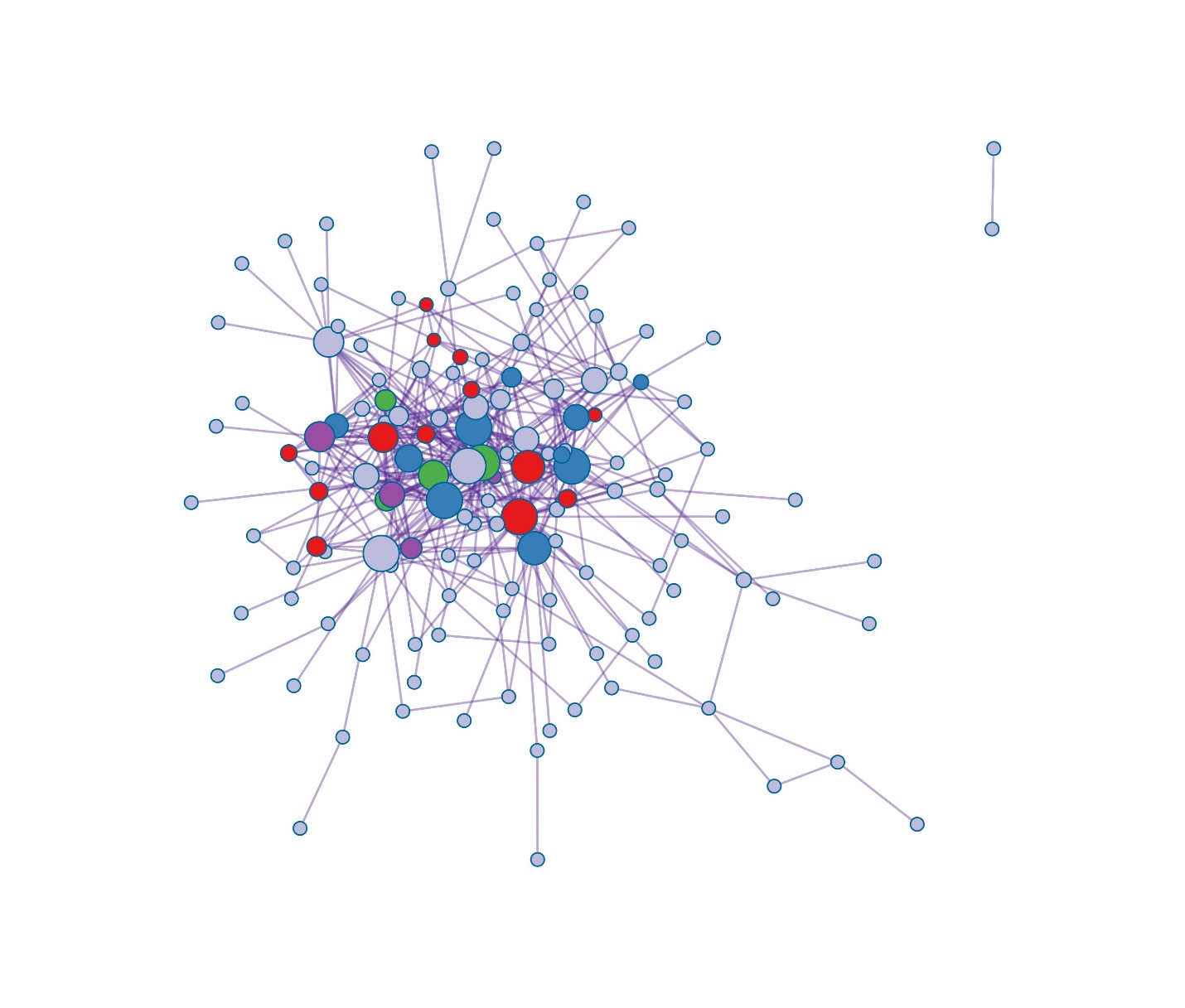


**Figure S4.** The Molecular Complex Detection (MCODE) algorithm with densely connected network components of gene lists for each MCODE clusters.
